# Contact with the baby following stillbirth and parental mental health and well-being: a systematic review

**DOI:** 10.1136/bmjopen-2015-008616

**Published:** 2015-11-27

**Authors:** Julie M Hennegan, Jane Henderson, Maggie Redshaw

**Affiliations:** National Perinatal Epidemiology Unit (NPEU), University of Oxford, Oxford, UK

**Keywords:** stillbirth, bereavement, touch, infant contact, systematic review

## Abstract

**Objective:**

To collate and critically appraise extant evidence for the impact of contact with the stillborn infant on parental mental health, well-being and satisfaction.

**Design:**

Systematic review.

**Data sources:**

A structured systematic search was conducted in 13 databases, complemented by hand-searching.

**Study eligibility criteria:**

English language studies providing quantitative comparison of outcomes for parents who held their baby or engaged in other memory-making activities, such as having photos and handprints, compared to those who did not, were eligible for inclusion.

**Outcome measures:**

Primary outcomes included clinically diagnosed mental health issues, standardised assessment of mental health issues or self-reported psychological distress. Secondary outcomes included poor health, relationship difficulties and satisfaction with the decision to have contact with the baby.

**Results:**

Two authors independently screened abstracts, selected potentially eligible studies, extracted data and evaluated the quality of included papers. 11 eligible studies, reported in 18 papers, were included. Studies were heterogeneous, precluding quantitative synthesis, thus a narrative synthesis is presented. Studies presented high risks of bias, particularly in regard to sample representativeness, and confounder identification and adjustment. Results were mixed concerning the impact of holding the stillborn baby on mental health and well-being. One study found no significant effects, and two studies reported no impact on depression. Conflicting effects were found for anxiety and post-traumatic stress. Other memory-making activities were not found to have a significant association with mental health or well-being outcomes. Across studies, mothers were satisfied with their decision to hold their baby or engage in other memory making.

**Conclusions:**

Evidence for the impact of holding the stillborn baby on mental health and well-being is sparse, and of poor quality. High-quality research guided by a priori hypotheses, with attention to potential confounders and moderating effects, is needed to provide more rigorous evidence to guide practitioners’ and parents’ decision-making for care following stillbirth.

**Review protocol number:**

PROSPERO CRD42014013890.

**Strengths and limitations of this study**

- This review is the first to collate and critically appraise extant evidence for the impact of contact with the stillborn infant on parental mental health, well-being and satisfaction.
- Sparse, conflicting evidence was found for the impact of holding the stillborn baby on maternal mental health and well-being, in the short and long term.
- Systematic searching identified no studies addressing the impact of contact with the stillborn baby on partners’ mental health outcomes.
- Included studies consistently suggested that mothers were satisfied with their decision to hold the stillborn baby.
- While this review provides quality appraisal of included studies, risk of bias assessment for non-randomised studies remains controversial and consensus for quality appraisal items is yet to be reached.

## Introduction

Stillbirth occurs in around 1 in 200 pregnancies in high-income countries.^1^ Perinatal loss is a devastating and traumatic event for women and their partners. The grief and distress experienced by parents has been documented across qualitative and quantitative studies.^2–6^ Recent investigation of parents’ experiences in the UK has emphasised the importance of the management of stillbirth and provision of care at this difficult time, reporting that care providers had ‘only one chance to get it right’.^7^

Standard care for parents during and after stillbirth has varied over time. Traditionally, parents did not see or hold their baby, which was taken away immediately after birth.^8^ ^9^ This approach received considerable criticism, and policies were changed to support parents to have contact with their baby, make memories and generate mementoes.^9^ ^10^ Qualitative studies found this to be a positive change. Mothers and fathers have both reported that the opportunity to see and hold their baby, and assistance to create memories, was invaluable.^7^ ^11–13^ Despite support from the qualitative literature, quantitative data on the impact of contact with the baby after stillbirth are sparse, and mixed findings have been noted.^14^ This has led to confusion regarding the best evidence-based care for parents in this situation. Clinical guidelines have differed in the recommendations provided. The National Institute for Health and Care Excellence (NICE) 2007 guidelines were criticised for recommending that mothers ‘should not be routinely encouraged to see and hold the dead infant’.^7^ ^10^ ^13–16^ The most recent guidelines^17^ have removed this recommendation, rather stating that the option to see, hold and have mementos of the baby should be discussed with women and facilitated by practitioners. Similarly, the Royal College of Obstetricians and Gynaecologists^18^ noted mixed evidence for the impact of holding the stillborn baby but recommends supporting the wish to do so when expressed, and, in Australia, Queensland Maternity and Neonatal Clinical Guidelines^19^ advise offering parents and relatives the option to hold their baby after stillbirth. To date, guidelines have been based on mixed qualitative and quantitative evidence.^14^ ^17^ Extant evidence for the effects of holding the stillborn baby has yet to be collated, and the quality of this evidence appraised. This review seeks to fill this gap, and to appraise and summarise current knowledge regarding the impact of holding the stillborn baby, and of additional memory-making activities.

### Outcome and moderator selection

One source of disparity between qualitative and quantitative reports suggesting negative effects of maternal contact with the stillborn baby may be the outcomes considered. Qualitative reports have typically focused on satisfaction, feelings of connectedness, and the emotional experience of mothers and fathers,^12^ ^13^ whereas quantitative studies have assessed either short or long-term distress manifested in anxiety and mental health issues.^20–22^ All of these psychological, satisfaction and parental preference outcomes are critical to consider in presenting parents with high-quality evidence with which to make an informed choice about their care,^23^ and in the development of clinical guidelines. Similarly, short-term and long-term outcomes must be considered in generating a more complete understanding of the impact of infant contact over time.^3^

Further, studies have suggested that characteristics such as the way the stillbirth was managed, characteristics of the baby, or maternal characteristics, may impact the association between contact and outcomes. Cacciatore *et al*^22^ found that, for women who were currently pregnant, having held their previous stillborn infant was associated with increased anxiety, but that this effect was reversed for women who were not currently pregnant. Subsequent live births may also moderate any long-term effects, although this has not been investigated.

The gestation of the stillborn baby has been identified as influencing both whether parents had contact with the baby, as well as the association between contact and outcomes.^22^ ^21^ Such differences may be attributable to a range of factors including attachment to the unborn child, staff expectations regarding contact and the condition of the baby. Indeed, the physicality of the baby who was stillborn has been identified in qualitative research as a significant concern for parents,^13^ and may itself moderate effects. Thus the condition of the baby, reasons for fetal death (eg, congenital anomaly) and the time from fetal death to delivery, could all be hypothesised to affect the short-term and long-term distress that may be caused by holding the stillborn baby. Finally, consistent with parents’ emphasis on the importance of care provider support in all aspects of stillbirth,^6^ the way in which contact is facilitated by staff may be an important aspect of the experience.^14^ An online survey of 840 mothers found that women felt more comfortable and less fearful when the infant was given to them to hold as a normal part of the birth process, in contrast to mothers who were first asked if they wanted to hold the baby.^24^ Staff support and facilitation of having time to hold the baby, and in assisting the collecting of mementoes, may all contribute to the experience at this critical time.^7^

### Past reviews

Koopmans *et al*^14^ conducted a systematic review of support practices for parents after perinatal death, including contact with the stillborn baby. As their eligibility criteria included randomised controlled trials (RCTs) only, no studies were included. RCTs are the most rigorous and, if well conducted, present the lowest risk of bias. However, RCTs could not ethically be used to assess the impact of holding the stillborn baby, or of memory-making activities. Thus the present review used more inclusive criteria to present an evaluation of best available evidence.

### Objectives

The purpose of this review was to collate and critically appraise extant evidence for the impact of contact with the stillborn infant on parental mental health, well-being and satisfaction. Further, this review seeks to highlight moderating factors, drawn from the literature, that may influence the relationship between contact with the infant and outcomes.^21^ ^22^ ^24^

## Methods

The protocol for this review was registered on PROSPERO (CRD42014013890) and is available online (https://www.npeu.ox.ac.uk/listeningtoparents). The Meta-analysis Of Observational Studies in Epidemiology (MOOSE) Group guidelines^25^ and Preferred Reporting Items for Systematic reviews and Meta-Analyses (PRISMA) statement^26^ were used to guide the review and reporting.

### Inclusion criteria

The primary intervention of interest was whether parents held their stillborn baby in the hours or days after birth. Rates of seeing the stillborn infant were recorded in data extraction, but were anticipated to be near universal, and so outcomes between these groups were not compared. Other activities that parents may undertake to build memories were included as secondary interventions. The selection of holding the baby as the primary intervention reflects the primacy of this practice in debate and controversies surrounding clinical guidance,^13–19^ and a preliminary review of the literature revealed that most studies focused on comparisons of holding.

Studies were included if they quantitatively compared outcomes for women or their partners who held or did not hold their stillborn baby. Studies were also included if they reported on other memory-making activities. RCTs, and prospective and retrospective cohort and case–control studies were eligible, as were cross-sectional studies. We included studies of all women and partners who had a singleton or multiple stillbirths. The definition of stillbirth differs across jurisdictions, with lower thresholds varying from 18 to 28 weeks’ gestation.^27^ In at least one prominent study of stillbirth contact and outcomes, a lower bound of 18 weeks was employed.^20^ It was not expected that first trimester loss (<13 weeks) would be defined as stillbirth in any studies of infant contact, thus this study was excluded. Beyond this very low threshold, we prioritised inclusivity in the review and studies were not excluded based on the gestational definition of stillbirth. Gestation was noted in study data extraction and considered an important moderator of effects. Recognising that studies of infant holding and contact may also include neonatal deaths, where >75% of the sample were stillbirths, studies were eligible for inclusion, and the proportion and outcomes included for parents who had a neonatal death were noted.

### Primary outcomes

Mental health was the primary outcome of interest, presented by level of measure validity.

1. Clinically diagnosed mental health issues, for example, depression, anxiety, post-traumatic stress disorder (PTSD)
2. Standardised assessment of mental health issues, for example, Edinburgh Postnatal Depression Scale (EPDS), Beck Depression Inventory, Depression Anxiety Stress Scales
3. Self-reported poor mental health or symptoms of psychological distress

### Secondary outcomes

Secondary outcomes reflected more general measures of well-being and relationships. Satisfaction with the contact decision was also included as a secondary outcome.

1. Self-reported measures of poor maternal/partner health identified by stakeholder user groups:^28^
  - Poor physical health
  - Fatigue or severe tiredness
  - Sleep problems
2. Personal relationship difficulties
3. Satisfaction with contact decision including:
  - Satisfaction with decision to hold or not to hold the stillborn baby
  - Satisfaction with the decision to have each additional memory-making activity (eg, satisfaction with decision to bathe baby)

### Search methods

The search strategy was developed based on inclusion criteria. In addition, the search strategies reported in a recent qualitative synthesis^6^ were consulted, as was the *Cochrane Pregnancy and Childbirth Group* specialised register MeSH terms.

No restrictions were set by date, publication type or language, although resource constraints meant that translation was only available for a limited range of languages. Searches were conducted in July 2015.

The following databases were searched from inception to present:

- Applied Social Science Index and Abstracts (ASSIA)
- British Nursing Index (BNI)
- Cochrane Database of Systematic Reviews
- Cochrane Pregnancy and Childbirth Group Trial Register
- Cumulative Index to Nursing and Allied Health (CINAHL) plus
- EMBASE
- Health Services Research Projects in Progress (HSRProj)
- MEDLINE
- Open Grey
- PsycINFO
- ProQuest Dissertations and Theses
- Science Citation Index
- Social Sciences Citation Index

The search strategy for MEDLINE is displayed in box 1 and was adapted for the other databases.

Box 1 **Search strategy for Medline**

**Search #1:** Exp stillbirth/OR exp fetal death/OR abortion, spontaneous/OR perinatal mortality/OR (‘fetus death’ OR ‘fetus loss’ OR ‘foetal loss’ OR ‘foetal death’ OR ‘fetal death’ OR ‘fetal loss’ OR ‘neonat* death’ OR ‘neonatal loss’ OR ‘newborn death’ OR ‘newborn loss’ OR ‘perinatal death’ OR ‘perinatal loss*’ OR ‘pregnanc* loss*’ OR ‘stillb*’ OR ‘still born’ OR ‘still birth)’.mp.

**Search #2:** Exp maternal behavior/OR paternal behavior/OR touch/OR rooming-in care/OR

(contact OR held OR hold* OR touch* OR bath OR bathing OR ‘care practice*’ OR ‘care guideline*’ OR footprint* OR handprint* OR memory OR memories OR momento OR photograph* OR policy OR policies OR ‘psychosocial care’ OR wash OR washing).mp.

**Search #3:** Exp adaptation, psychological/OR exp anxiety/OR exp anxiety disorders/OR Exp depression/OR exp depression postpartum/OR depressive disorder/OR Exp mood disorders/OR exp grief/OR Exp mental health/OR mental disorders/OR Adjustment Disorders/OR exp stress disorders, post-traumatic/OR stress, psychological/OR sleep disorders/OR ‘sleep initiation and maintenance disorders’/OR (adjustment OR anxi* OR coping OR depress* OR distress OR divorce OR fatigue OR ‘interpersonal difficult*’ OR ‘interpersonal problem*’ OR ‘interpersonal trouble*’ OR ‘insomnia’ OR ‘mental health’ OR ‘mental disorder’ OR ‘mental illness’ OR ‘physical health’ OR ‘poor sleep’ OR ‘posttraumatic stress’ OR ‘post-traumatic stress’ OR ‘postnatal anxiety’ OR ‘postnatal depression’ OR ‘postpartum anxiety’ OR ‘postpartum anxiety’ OR ‘puerperal depression’ OR ‘puerperal depression’ OR psychological OR psychosocial OR PTSD OR regret* OR ‘relationship break*’ OR ‘relationship difficult*’ OR ‘relationship dissolution’ OR ‘relationship problem*’ OR ‘relationship trouble*’ OR satisfaction OR satisfied OR stress* OR ‘sleep problem*’ OR ‘sleep difficult*’ OR ‘sleeping problem*’ OR ‘sleeping difficult*’ OR tired* OR wellbeing OR ‘well being’ OR ‘well-being)’.mp.

**Search #4:** Search #1 AND Search #2 AND Search #3

### Searching other resources

1. Additional grey literature from the websites of the Stillbirth and Neonatal Death Charity (Sands) and the International Stillbirth Alliance
2. The reference lists and forward citations of all studies meeting inclusion criteria
3. Subject experts were contacted to identify unpublished or ongoing research

### Study selection, appraisal and synthesis

Titles and abstracts returned from searches were independently screened by two reviewers (JMH and JH), who also independently screened full-text articles. Where there was disagreement, reviewers met to reach consensus, and studies were referred to a third reviewer (MR).

While there are clear guidelines for assessing the risk of bias in RCTs,^29^ risk of bias assessment for observational research remains controversial.^30^ The risk of bias and quality of included studies were assessed for this review using a checklist developed based on the items of the STrengthening the Reporting of OBservational studies in Epidemiology (STROBE) statement,^31^ Critical Appraisal Skills Program^32^ ^33^ checklists for cohort and case–control trials, and the Newcastle-Ottawa Scales (NOS) for assessing the quality of non-randomised studies,^34^ based on recent appraisal of quality assessment tools for observational studies.^30^

The following items were appraised for each study and rated as high, low or unclear risk of bias:

- Sample representativeness;
- Adequacy of exposure measurement (ie, whether the parents held the stillborn baby or engaged in other memory-making activities);
- Incomplete outcome data (attrition bias);
- Selective outcome reporting;
- Other bias.

Two additional items were appraised to assess group comparability and statistical adjustment for potential confounders:

- Comparability of exposed and non-exposed participants;
- Adequacy of statistical methods and confounder adjustment.

These two items were rated as high risk (little comparability/no adjustment), moderate risk (comparable/adjusted on demographic characteristics only) or low risk (comparable/adjusted on more specific characteristics for these comparisons, eg, pre-pregnancy mental health issues, mode of delivery). This rating was used to provide a more nuanced appraisal of study quality and is consistent with recent evidence that the results of non-randomised designs more closely approximate those of randomised studies when adjustment is made for relevant area-specific characteristics, rather than for characteristics of convenience such as demographics.^35^

For each study, two reviewers (JMH and JH) independently appraised study quality. Disagreement was resolved through discussion and referral to the third reviewer (MR). Data were extracted independently by two reviewers.

### Data synthesis

Narrative synthesis is presented for included studies as heterogeneity in participants and methods precluded quantitative synthesis.^26^ ^29^ Standardised measures of effect (ORs, standardised mean differences) were calculated to aid comparison. Where studies had adjusted for potential confounders, adjusted measures of effect are included in preference to crude comparisons. Where outcomes are reported for multiple time points, data are presented for each time point. In addition, measures of effect for key subgroups/proposed moderators (see below) are presented. Standardised measures of effect not reported in studies and calculated by review authors are denoted by footnotes in the tables. Effect sizes were calculated using the Campbell Collaboration online effect size calculator,^48^ employing study frequencies, means, SD and correlation coefficients. Effect estimates for outcomes compared between those who held and did not hold their stillborn baby, and outcomes compared for other types of contact and memory-making activities are presented separately.

### Subgroup comparisons

From a review of the background literature, the following characteristics were hypothesised to influence the association between contact with the stillborn baby and outcomes:

1. Timing of maternal/paternal outcome assessment since the stillbirth;
2. Women pregnant at the time of outcome assessment;
3. Subsequent live birth/s;
4. Gestation of stillbirth;
5. Reason for stillbirth, for example, congenital abnormality or other causes;
6. Time from antepartum death to birth;
7. Level of support for contact/memory making provided by staff;

Where possible, outcomes were stratified according to these subgroups/moderators, and changes to relationships between contact and outcomes noted.

## Results

**Figure 1:**
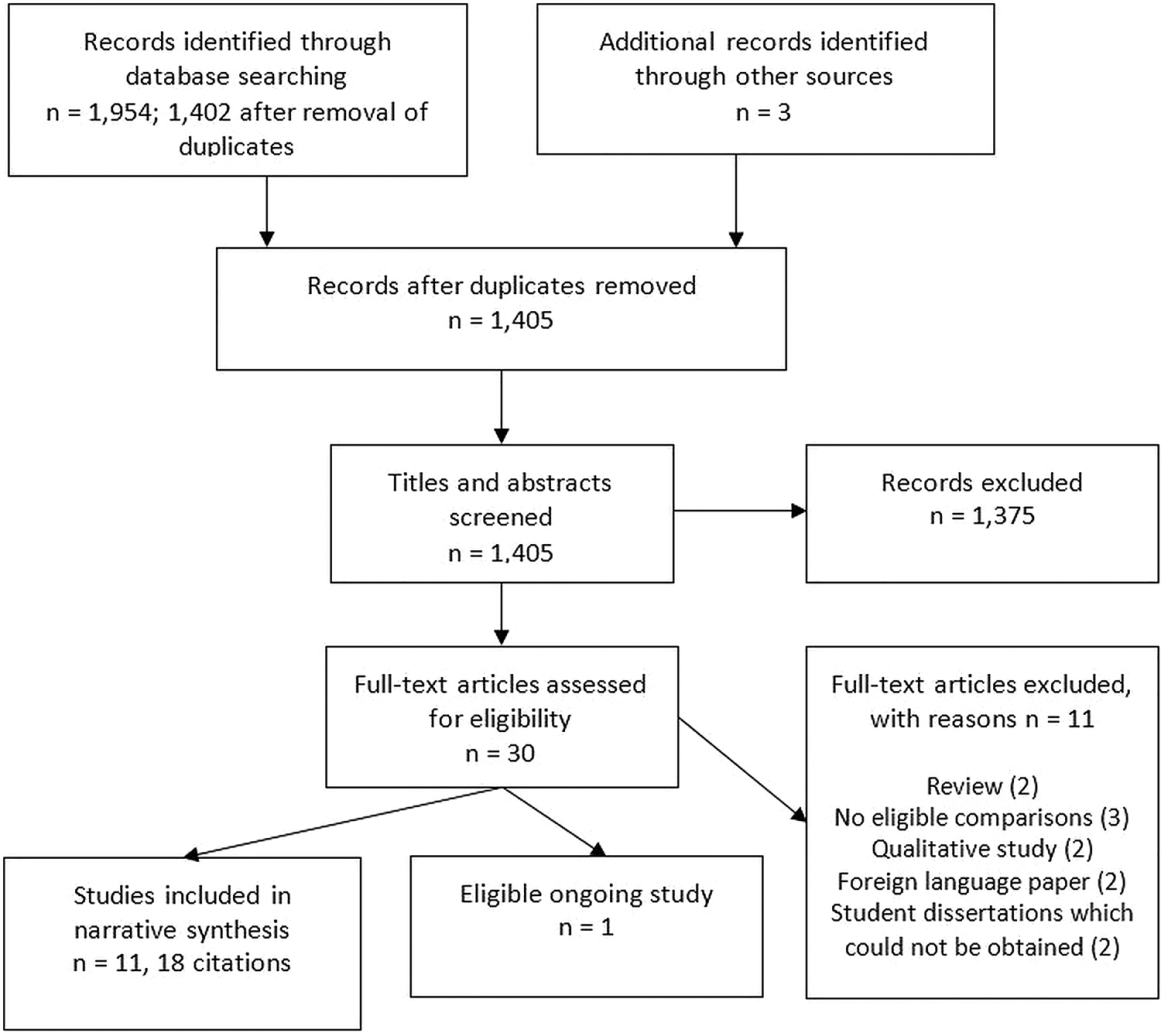
Study flow chart.

A total of 1402 unique papers were identified and screened. Figure 1 displays the searching flow chart and reasons for exclusions.^26^

### Description of studies

Eleven studies reported in 18 papers were eligible for inclusion. This included 16 peer-reviewed publications and two unpublished dissertations.^47^ ^39^ One conference abstract was identified providing evidence of an eligible study. The authors were contacted but results and full study detail were unavailable at the time of writing, thus the study was listed as eligible but ongoing.^49^

The characteristics of the 11 studies included are displayed in table 1.

**Table 1:** Characteristics of included studies

Study ID	Study type	Date data collected	Location/setting	Time since stillbirth	N	Inclusion criteria	Stillbirth gestations
Bennett *et al*^36^	Cross-sectional retrospective telephone survey	2007	Four hospitals in the Boston area, USA	0–5 years Mean=35 months, SD=20	55	Women identified by maternity care providers as eligible for inclusion. Women <18 years who lost a child to SIDS or had an elective abortion were not recruited	Perinatal loss (from 20 weeks’ gestation to 1 month postpartum) Mean=28 weeks’ gestation
	Type of contact assessed		Holding the infant. Taking photos of the infant				
	Eligible outcomes assessed		Perinatal grief scale (PGS^50^); Inventory of Complicated Grief (ICG^51^); PTSD (post-traumatic stress disorder) Checklist (PCL^52^); Brief Symptom Inventory 18 (BSI^53^). Satisfaction with decision to hold baby				
Blood and Cacciatore^46^	Cross-sectional retrospective online survey	October 2011–April 2012	Primarily US participants	1–54 years (75% within past 6 years)	123	Study included parents of children who had died. Mothers and fathers both included (96% female respondents)	36 ‘late miscarriage’ (15–26 weeks) 87 ‘stillborn or perinatal death’ (27 weeks to 6 days)
	Type of contact assessed		Post mortem photography				
	Eligible outcomes assessed		Satisfaction with decision to have post mortem photography				
Cacciatore *et al*^22^	Cross-sectional retrospective online survey	February 2004–September 2005	USA (72%), UK (11%), Australia (9%), Canada (5%)	<1–3+ years <1 years 51% 1–2 years 15% 2–3 years 9% >3 years 25%	2292	Volunteers recruited from relevant organisation websites and forums	From 20 weeks’ gestation Third trimester loss 79.5% of the sample
	Type of contact assessed		Holding, dressing/washing the stillborn infant				
	Eligible outcomes assessed		Anxiety and depression (25-item Hopkins Symptom Check List, HSCL^54^). Satisfaction with decision to hold baby				
Crawley *et al*^37^	Cross-sectional retrospective online survey	February 2010–July 2010	UK	0–10 years Mean=27.9 months, median=18.5 months	162	Women who were at least 18 years old and gave birth in the UK in the past 10 years to a stillborn baby of at least 20 weeks gestation	From 20 weeks gestation Mean=35.4 weeks gestation, median=38, range=20–43 weeks
	Type of contact assessed		Making memories (analyses by ‘number of memory-making activities’). Activities included: seeing baby, holding, naming, holding a funeral, creating memory box, taking photos, scattering ashes, family seeing baby, hand/footprints, taking a lock of hair, dressing the baby, others seeing the baby, bathing the baby, taking the baby home				
	Eligible outcomes assessed		Depression and anxiety in the past month (Depression, Anxiety and Stress Scale; DASS-21^55^). PTSD symptoms in the last month (Post-traumatic Stress Symptom Scale, PTSS^56^)				
Fink^47^	Cross-sectional retrospective online survey	March 2008–December 2008	Primarily US participants	0–2 years	498	Women at least 18 years of age who had experienced a stillbirth in the past 2 years	Not reported
	Type of contact assessed		Given the opportunity to hold the baby (actual holding not reported). Memory box or received other mementos				
	Eligible outcomes assessed		Perinatal Grief Scale (PGS^50^)				
Gravensteen *et al*^38^	Cross-sectional retrospective postal survey	2008–2009	Norway (women from 2 hospitals)	5–18 years	101	Women who had a verified diagnosis of stillbirth (≥23 weeks’ gestation or ≥500 g) in a singleton or twin pregnancy between 1 January 1990 and 31 December 2003	From 23 weeks’ gestation
	Type of contact assessed		Holding the infant. Other memory-making activities assessed and proportion engaged in, reported by number of comparisons conducted (activities included: photographs, hand/footprint, naming, memorial or funeral, having the baby buried in a grave)				
	Eligible outcomes assessed		Post-traumatic Stress Symptoms (Impact of Event Scale, IES^57^); subjective well-being (General Health Questionnaire; GHQ-20^58^); depression (Centre for Epidemiological Studies Depression Scale; CES-D^59^). Satisfaction with decision to hold baby				
Hughes *et al*^2^ ^3^ ^20^ ^39^	Longitudinal (although still retrospective with regard to holding), interview and survey	Time 1: not reported Time 2: not reported Time 3: October 2003–July 2006	UK (women from 3 district general hospitals)	T1: 10 months–5 years (Median gap between loss and expected delivery date 18.5 months) T2: 1 year after subsequent live birth T3: 6–8 years after subsequent live birth	T1: 65 T2: 55 T3: 52	Pregnant women (who had subsequent live birth), who had no previous live children Excluded women in treatment for physical or mental illness and those whose stillbirth was the result of elective termination for abnormality	From 18 weeks’ gestation
	Type of contact assessed		Holding the infant. Other contact assessed but comparisons not reported.				
	Eligible outcomes assessed		Postnatal depression (Edinburgh Postnatal Depression Scale, EPDS^60^); anxiety (state scale from the Spielberger State-Trait Anxiety Inventory, STAI^61^), Depression (Beck Depression Inventory^62^); Post-traumatic stress (PTSD-1 interview^63^); Marital satisfaction; Psychological symptoms (DSM-III CID) (T3).				
Kuti and Ilesanmi^40^	Cross-sectional retrospective interviewer-administered questionnaire	January–June 2009	Nigeria (University teaching hospital)	6 months–16 years	45	Women registered for prenatal care who had a previous stillborn infant	Not explicitly reported (Nigerian definition of stillbirth: >1 kg or 28 weeks’ gestation)^64^
	Type of contact assessed		Holding the infant. Other memory-making activities including: taking photos, obtaining mementos, naming infant.				
	Eligible outcomes assessed		Maternal self-assessment of ‘recovery’ from stillbirth. Satisfaction with decision to see/hold infant.				
Lasker and Toedter^9^	Longitudinal interview and survey	1984–1989	Pennsylvania, USA	Interviews at T1: 2 months T2: 1 year T3: 2 years following loss	138 mothers (56 partners)	Women who had attended public or private service provider and who had experienced a pregnancy loss or neonatal death 55 women had a stillbirth	From 16 weeks’ gestation
	Type of contact assessed		Holding the infant. Other memory making activities including: taking and keeping pictures, holding a funeral or memorial service				
	Eligible outcomes assessed		Perinatal Grief Scale (PGS^50^). Satisfaction with decision to hold infant and care received.				
Rådestad *et al*^21^ ^41–44^	Cross-sectional retrospective nationally representative postal questionnaire	October–November 1994	Sweden	3 years post-stillbirth	314	Women who had a singleton stillbirth in Sweden in 1991	From 28 weeks’ gestation
	Type of contact assessed		Holding the infant. Other memory-making activities included: time with the baby, kissed/caressed, dressed the baby, had photo taken, kept token of remembrance				
	Eligible outcomes assessed		Anxiety (assessed using both state and trait aspects of the Spielberger State-Trait Anxiety Inventory, STAI^61^); Depression (Centre for Epidemiological Studies Depression Scale, CES-D^59^), a range of physical symptoms were also assessed (see table 1)				
Rådestad *et al*^45^	Cross-sectional retrospective postal questionnaire	2001	Stockholm, Sweden	3 months after discharge	33	Nordic-born women who had a singleton stillbirth	From 22 weeks gestation 22–28 weeks 35%, 29+ weeks 65%
	Type of contact assessed		Holding the infant				
	Eligible outcomes assessed		Fear, regret, tenderness, warmth, pride, insecurity, discomfort, grief assessed on a Likert-scale from 0 (not at all) to 4 (very much)				

Studies were published between 1994 and 2014. The majority of studies were conducted in the USA and the number of participants ranged from 45 to 2292, for a total of 3826 participants across all studies with a median of 123. Designs were primarily cross-sectional retrospective surveys. Studies varied in the primary research question. Some focused on the impact of contact with the infant while others assessed multiple predictors of mental health or well-being, with infant contact included as one of these predictors.

Nine studies included comparisons of the primary intervention (holding the baby) and nine comparisons of secondary (memory making) activities. All studies relied on women's self-report to assess contact with the baby. No studies reported the type (eg, skin-to-skin contact), timing or duration of holding. The proportion of women reporting that they held their stillborn baby varied from 0% in Nigeria, 40 to 94% of women in Sweden.^21^ Older studies reported lower rates of holding, for example, Lasker and Toedter^9^ 42% and Crawley *et al*^37^ 93%.

The timing of outcome assessment varied from a few months after the stillbirth to many years later (54 years in one study^43^). Some studies reported outcomes at a single time point and some did so at multiple time points. Outcomes were typically collapsed across time periods. Gestation of the baby at delivery varied across studies, as did gestation at which the death was defined as stillbirth rather than a miscarriage. Gestations of 16 weeks were included in some studies, including those by Blood and Cacciatore,^46^ and Lasker and Toedter,^9^ while others used a 20 or 22 week boundary, and yet others, a later 28 week criterion, including studies by Rådestad and co-authors^21^ ^41–44^ and Kuti and Ilesanmi.^40^

Two studies presented comparisons including perinatal loss of up to 6 days, 46 or 1 month, 36 although the majority of both samples were stillbirths.

Most studies assessed one or more mental health outcomes including depression, anxiety and post-traumatic stress symptoms through standardised scales such as the Perinatal Grief Scale (PGS^50^), Impact of Event Scale (IES^57^) and the Centre for Epidemiological Studies Depression (CES-D^59^) Scale. Many studies also investigated the secondary outcome of women's satisfaction with their decision to hold or have other contact with the baby, typically measured through a study-specific, single Likert-scale item. Fewer studies included other secondary outcomes such as physical symptoms or relationship difficulties.

### Quality of included studies

An assessment of the strengths and weaknesses of included studies is essential in estimating the reliability of effect sizes presented.^65^ Risk ratings for aspects of study quality are presented in table 2. Further detail and support for study ratings are provided in online supplementary materials.

**Table 2:** Risk of bias in included studies

Study ID	Sample representa tiveness*	Adequacy of exposure measurement†	Completeness of outcome data‡	Selective outcome reporting	Other bias	Comparability of exposed and non-exposed participants	Adequacy of statistical methods and confounder adjustment
Bennett *et al*^36^	High	Low	Low	Unclear	–	Unclear	High
Blood and Cacciatore^46^	High	Low	Low	Unclear	–	Unclear	High
Cacciatore *et al*^22^	High	Low	Low	Unclear	–	High	Low
Crawley *et al*^37^	High	Low	Low	Unclear	High	Unclear	Moderate
Fink^47^	High	High	Low	Unclear	–	Unclear	High
Gravensteen *et al*^38^	High	Low	Low	Unclear	–	Unclear	Moderate
Hughes *et al*^2^ ^3^ ^20^ ^39^	High	Low	Low	High	–	Unclear	High
Kuti and Ilesanmi^40^	High	Low	Low	Unclear	–	Unclear	High
Lasker and Toedter^9^	High	Low	Low	Unclear	High	Unclear	High
Rådestad *et al*^21^ ^41–44^	Low	Low	Low	Unclear	–	Moderate	Low
Rådestad *et al*^45^	High	Low	Low	Unclear	–	Unclear	High

*Reflected through adequate recruitment, exclusion criteria, response rates and comparability to wider birthing or stillbirth population.

†Adequacy of the assessment of contact with the stillborn infant. ‡Attrition bias.

#### Sample representativeness

Sample representativeness reported a ‘high risk of bias’ across most studies. Four studies^22^ ^37^ ^46^ ^47^ used volunteer samples from stillbirth organisation mailing lists that are likely to represent a much more actively engaged sample. Where sample demographics were compared to the broader birthing population, participants were typically younger and more highly educated. Four studies recruited women from hospitals or birthing centres.^9^ ^36^ ^38^ ^40^ Reported response rates were often low (Bennett 16%; Gravensteen 31%). The study by Hughes and co-authors^2^ ^3^ ^20^ ^39^ only included participants who were currently pregnant (time 1) with no previous live children, who went on to have a live birth, and were not in treatment for mental or physical health reasons. Generalisability is highly limited by the specific nature of this group. Only one study (Rådestad and co-authors^21^ ^41–44^) was considered low risk of bias—a nationwide study of all mothers who had a stillbirth, with a high response rate of 83%.

#### Adequacy of exposure measurement

All studies assessed contact with the infant through women's retrospective self-report. Given the salience of stillbirth in women's lives, this was considered a reliable and adequate exposure measurement. One study reported whether mothers were given the opportunity to hold their stillborn baby (rather than if they did so).^47^ No studies clearly defined ‘holding’, and none assessed the timing, type or duration of contact with the baby; doing so could have provided important additional information.^66^

#### Incomplete outcome data

As the majority (9/11) of studies were cross-sectional, there was no risk of attrition. In the two longitudinal studies, retention rates were high, representing a low risk of attrition bias.

#### Selective outcome reporting

Hypotheses and analyses were not pre-specified in any of the studies. It is not possible to determine if measures reported in Methods section represent all data that were collected or only those data reported in Results section. It is therefore unclear whether other results or analyses were excluded. Given the mixture of both positive and negative results, and lack of studies reporting no association, it is probable that selective publication has occurred.

Selective outcome reporting was considered a high risk in one study. Hughes and co-authors^2^ ^3^ ^20^ ^39^ reported (in text) that corrected analyses for the effect of time since the stillbirth and socioeconomic status revealed the association between infant contact and depression to no longer be significant. However, the figures on which this was based were not reported, and the role of time since the stillbirth and socioeconomic status were not reported in the follow-up at time 3.

#### Other bias

Two studies were classified as ‘high risk’ of additional bias.^9^ ^37^ For both studies, this was due to the amalgamation of different types of infant contact, memory-making and other interventions after stillbirth. Analyses in both studies were restricted to this combined predictor variable and thus failed to test the impact of any individual intervention. Combining interventions was analytically inappropriate, demonstrating a lack of theory regarding how or why each intervention might positively or negatively affect mental health and well-being. It is also possible for such strategies to mask selective outcome reporting, where comparisons according to individual interventions were not significant.

#### Comparability of exposed and non-exposed

The majority of studies (9 of 11) failed to investigate the comparability of mothers who held, or did not hold, their baby. As this comparability was not assessed, studies were classified as ‘at unclear risk of bias’ (see table 2). However, as groups were not randomly assigned it is highly likely that in all of these studies the groups are not comparable. Cacciatore *et al*^22^ contrasted demographic as well as study-specific characteristics, and found that exposed and non-exposed participants differed in the timing of their loss, time since the loss and primary ethnicity. Rådestad and co-authors^21^ ^41–44^ also contrasted groups on a number of demographic and study-specific factors, and found that the groups did not differ according to maternal or baby characteristics (p.424), but did differ in maternal education. This was rated as at a ‘moderate risk of bias’.

#### Adequacy of statistical methods and confounder adjustment

Seven studies were considered to have a high risk of bias, primarily due to failure to adjust for potential confounders in the relationship between holding the infant, and mental health and well-being outcomes. Most studies presented only univariate analyses (see tables 3 and 4). Cacciatore *et al*^22^ and Rådestad and co-authors^21^ ^41–44^ were rated as ‘low risk of bias’ for confounder adjustment, as differences between the groups were evaluated and any significant factors included in multivariable models for adjustment. Both Crawley *et al*^37^ and Gravensteen *et al*^38^ included analyses that focused on predictors of post-traumatic stress, and adjusted for many demographic and study-specific factors that differed between those considered to have high levels of Post-traumatic Stress Symptom Scale (PTSS)/PTSD and those who did not. In doing so, these studies adjusted for many potential confounders, but as differences between exposed and non-exposed participants were not assessed, it is possible that important differences were missed. These studies were considered to have a moderate risk of bias.

**Table 3:** Impact of holding the stillborn baby on primary and secondary outcomes

Study ID	N	Time since stillbirth	Outcomes assessed	Measure of effect*	Adjustment for
Bennett *et al*^36^	55	0–5 years, mean=35 months	Complicated Grief (ICG)	d=−0.02 (−0.66 to 0.62)†	None
			PTSD symptoms (PCL)	d=0.17 (−0.47 to 0.81)†	
			Depression/anxiety (combined PGS, Brief Symptom Inventory)	d=0.17 (−0.47 to 0.81)†	
			Satisfaction with decision to hold baby	Of the 78% of the sample who held their baby, 85% of women reported this to be extremely helpful	
Cacciatore *et al*^22^	2292	<1–3+ years <1 years 51% 1–2 years 15% 2–3 years 9% >3 years 25%	Anxiety (HSCL)	Not currently pregnant: OR 0.68 (0.49 to 0.95) Currently pregnant: OR 3.79 (1.42 to 10.1)	Gestation of stillbirth (by trimester) Time since loss
			Depression (HSCL)	Not currently pregnant: OR 0.72 (0.51 to 1.02) Currently pregnant: OR 2.13 (0.90 to 5.06)	
			Satisfaction with decision to hold baby	99.5% of 2035 mothers who held their baby were glad they did 8.2% of 226 mothers who did not hold their baby were glad they did not; 79.5% wished they had held their baby and 12.3% were ‘indifferent’	None
Crawley *et al*^37^	162	0–10 years, median 18.5 months	Depression in the past month (DASS-21)	Authors collapsed comparisons across holding the infant and memory-making activities (including photographs, hand/footprints, creating memory box) as a single variable. Authors reported no relationship between memory-making and mental health outcomes. Data not shown and proportions/effect sizes not reported	
			Anxiety in the past month (DASS-21)		
			PTSD symptoms in the past month (PSSS)		
Gravensteen *et al*^38^	101	5–18 years	Post-traumatic Stress Symptoms (IES) IES >20 vs <20 (20 was considered possible clinical case level)	OR 0.17 (0.05 to 0.56)	Maternal age, parity, induced abortion prior to stillbirth
			Satisfaction with decision to hold baby	86% of mothers who held their baby reported ‘it felt good’ 62% of the mothers who did not hold their baby regretted this decision	None
Hughes *et al*^2^ ^3^ ^20^ ^39^	T1: 65 T2: 55 T3: 52	T1: 10 months to -5 years (median 18.5 months) T2: 1 year after subsequent live birth T3: 6–8 years after subsequent live birth	Depression (EPDS >14)/(EPDS continuous) Depression (BDI, continuous)	T1: OR 4.18 (1.19 to 14.69)/d=0.48 (−0.009 to 0.98)‡ T2: d=0.42 (−0.12 to 0.96)‡	None
			Anxiety (STAI state >44)/(continuous)	T1: OR 2.67 (0.87 to 8.17)/d=0.51 (0.01 to 1.00)‡ T2: OR 3.83 (0.73 to 20.04)/d=0.43 (−0.10 to 0.99)‡	None
			PTSD-1 interview (diagnosis met)/(continuous)§ PTSD (DSM-IV SCID)	T1: OR 4.35 (0.84 to 22.63)/d=0.59 (0.05 to 1.09)‡§ T2: (not assessed)/d=1.0 (0.44 to 1.56)‡§ T3: d=0.78 (0.21 to 1.35)‡	None
			Marital separation	T3: OR 4.50 (1.23 to 16.49)‡	None
Kuti and Ilesanmi^40^	45	6 months to 16 years	Maternal self-assessment of ‘recovery’ from stillbirth	No mothers were given the opportunity to hold the baby and thus comparisons could not be conducted	
			Satisfaction with decision to hold infant	8 (17.8%) of women wished they had had the opportunity to hold their infant	
Lasker and Toedter^9^	138	T1: 2 months T2: 1 year T3: 2 years	Postnatal grief (PGS)	Postnatal grief outcome was only evaluated using a combined variable representing the total number of interventions, thus the individual impact of any single intervention cannot be determined	
			Satisfaction with decision to hold infant (time 1)	Early fetal death (16–28 weeks): no significant difference in satisfaction with decision Late fetal death (27+ weeks): women who held their baby significantly more satisfied with their decision than women who did not hold their baby (proportions not reported)	None, results split by gestation of stillbirth
Rådestad *et al*^21^ ^41–44^	314	3 years	Anxiety (STAI state)	28–37 weeks’ gestation: OR 0.70 (0.30 to 1.66) 37+ weeks’ gestation: OR 1.70 (0.34 to 8.62)	None (only education significantly differed between those who held and those who did not)
			Depression (CES-D) (dichotomous, scores above 90th centile)	28–37 weeks’ gestation: OR 0.50 (0.20 to 1.30) 37+ weeks’ gestation: OR—(Fisher's exact test, p=0.055)	
			Backache, stomach problems, headache, tachycardia, chest pressure, panic attacks, nausea or fainting, weakness, sleep disturbances, situation in home and family, situation at work, health, leisure time, physical fitness, appetite, temper, energy, patience, self-confidence	No significant differences with the exception of: 28–37 weeks’ gestation: stomach problems: OR 0.10 (0.02 to 0.94) 37+ weeks’ gestation: headache: OR 0.23 (0.06 to 0.96); sleep OR: 0.28 (0.13 to 0.60)	
Rådestad *et al*^45^	33	3 months	Fear, regret, tenderness, warmth, pride, insecurity, discomfort, grief	94% of 33 women held their baby When holding their baby, all mothers felt tenderness and grief; 94% warmth, 81% pride; 48% insecure, 39% discomfort and 35% fear. The mothers of stillborn babies born before 28 weeks’ gestation experienced more fear and insecurity when they held their baby, but differences were not statistically significant (proportions not reported)	None—follow-up comparisons according to gestation of stillbirth

*Where possible standardised mean differences (d) or ORs and 95% CIs were calculated.

†Calculated using study reported frequencies and correlations.

‡Calculated using study reported mean and SD for continuous outcomes, and study reported frequencies for dichotomous outcomes.

§Based on proportions reported in Hughes *et al*^20^ (proportions for time 1 and 2 PTSD differ between refs 3 and 20).

BDI, Beck Depression Inventory; CES-D, Centre for Epidemiological Studies Depression Scale; DASS-21, Depression, Anxiety and Stress Scale; DSM, Diagnostic and Statistical Manual of Mental Disorders; EPDS, Edinburgh Postnatal Depression Scale; HSCL, 25-item Hopkins Symptom Check List; ICG, Inventory of Complicated Grief; IES, Impact of Event Scale; PCL, PTSD Checklist; PGS, perinatal grief scale; PTSD, post-traumatic stress disorder; SCID, Structured Clinical Interview for DSM Disorders; STAI, Spielberger State-Trait Anxiety Inventor.

**Table 4:** Impact of other contact with the stillborn baby on primary and secondary outcomes

Study ID	N	Time since stillbirth	Outcomes assessed	Measure of effect*	Adjustment for
Bennett *et al*^36^	55	0–5 years, mean=35 months	Complicated grief (ICG)	Taking pictures of infant: d=−0.29 (−0.97 to 0.40)†	None
			PTSD symptoms (PCL)	Taking pictures of infant: d=−0.16 (−0.84 to 0.53)†	
			Depression/anxiety (combined PGS, BSI)	Taking pictures of infant: d=0.08 (−0.61 to 0.76)†	
			Satisfaction with decision to take pictures of the baby	Of the 82% of the sample who had photos taken of their baby, 75% reported this was extremely helpful	
Blood and Cacciatore^46^	123	1–54 years (75% within past 6 years)	Satisfaction with decision to have post mortem photography	Only 9 of 123 parents did not have post mortem photography. Only 1 of the 9 was content not having post-mortem photographs	None
Cacciatore *et al*^22^	2292	<1–3+ years <1 years 51% 1–2 years 15% 2–3 years 9% >3 years 25%	Anxiety (HSCL)	Dressed or washed baby: not currently pregnant: OR 0.88 (0.68 to 1.13) Currently pregnant: OR 1.04 (0.58 to 1.86)	Gestation of stillbirth (by trimester), Maternal age
			Depression (HSCL)	Not currently pregnant: OR 1.02 (0.80 to 1.30) Currently pregnant: OR 0.98 (0.55 to 1.76)	
			Satisfaction with decision to wash or dress baby	98.3% of 473 mothers who dressed or washed their stillborn baby were glad they did 12.4% of 1784 mother who did not dress or wash their baby were glad they had not. 22.1% were ‘indifferent’ and 65.5% wished they had	None
Crawley *et al*^37^	162	0–10 years, Median 18.5 months	Authors collapsed comparisons across holding the infant and memory-making activities (including photographs, hand/footprints, creating memory box) as a single variable. Authors reported no relationship between memory making and outcomes		
Fink^47^	498	0–2 years	Postnatal grief (PGS)	Being given the option to hold the baby was not significantly correlated with PGS (0.04, p>0.05) Bivariate correlations between contact and PGS: memory box: −0.14, p<0.01 Hand/footprints: −0.05, p>0.05 Pictures: −0.07, p>0.05 Lock of hair: −0.01, p>0.05 Memory box β=−0.11, p=0.018 in final stepwise regression model	Stepwise regression included: living children, race, pregnancy history, autopsy, hospital disposal, opportunity to talk about baby, clear communication from care providers
Gravensteen *et al*^38^	101	5–18 years	Post-traumatic Stress Symptoms (IES)	Having an arranged memorial was not significantly associated with IES scores OR 0.48 (0.16 to 1.40)	
Kuti and Ilesanmi^40^	45	6 months–16 years	Maternal self-assessment of ‘recovery’ from stillbirth	No mothers were given the opportunity have other contact with the infant	
			Satisfaction with other contact with the infant	2 (4.4%) of the women reported wished they had the opportunity to take photos of their infant	
Lasker and Toedter^9^	138	T1: 2 months T2: 1 year T3: 2 years	Postnatal grief (PGS)	Postnatal grief outcome was only evaluated using a combined variable representing the total number of interventions, thus the individual impact of any single intervention cannot be determined	
			Satisfaction with decision to have contact with infant (time 1)	Mothers who were given a picture of the baby were more satisfied with this decision, as were those who had a death certificate, and those who named the baby for both those who had an early or late fetal death (proportions not reported)	None, results split by gestation of stillbirth
Rådestad *et al*^21^ ^41–44^	314	3 years	Depression (CES-D) (dichotomous, scores above 90th centile)	Not being with the baby as long as wished: RR 6.9 (2.4 to 19.8) Did not kiss or caress baby: RR 2.1 (0.8 to 5.6) Did not dress the baby: RR 1.6 (0.8 to 3.3) Did not keep photo of baby: RR 0.9 (0.2 to 4.6) Did not keep token of remembrance: RR 1.2 (0.4 to 3.3)	Maternal education, employment and marital status

*Where possible standardised mean differences (d) or ORs and 95% CIs were calculated.

†Calculated using study reported frequencies and correlations.

BSI, Brief Symptom Inventory 18; CES-D, Centre for Epidemiological Studies Depression Scale; HSCL, 25-item Hopkins Symptom Check List; ICG, Inventory of Complicated Grief; IES, Impact of Event Scale; PCL, PTSD Checklist; PGS, perinatal grief scale; PTSD, post-traumatic stress disorder.

### Impact of contact with the stillborn baby

Eight studies provided comparisons of women who held and did not hold their baby (table 3). One study^37^ measured this type of contact but only provided analysis by a combined infant contact experience variable. We have retained this study and included reported outcomes, but effect sizes could not be calculated for comparison. Six studies provided comparisons assessing the impact of other contact or memory-making activities on outcomes. No studies reported on clinical diagnosis of mental health conditions, with most reporting on outcomes using standardised assessments. Results are presented below with primary mental health outcomes and secondary well-being outcomes presented together, with few studies reporting on these secondary well-being outcomes. Most studies reported on satisfaction with holding or having other contact with the baby. Results are presented first for the primary intervention of holding the baby after stillbirth, then secondary interventions including the range of other types of contact (such as bathing the baby).

### Holding the infant

#### Mental health and well-being

Five studies compared primary mental health outcomes for those who held or did not hold their baby. One study also provided assessment of secondary outcomes of general health, sleep disturbance, energy and self-confidence^21^ ^41–44^. One study, of 55 women, found no effect of holding the infant on complicated grief or PTSD symptoms, or on an anxiety/depression measure.^47^ Rådestad and co-authors^21^ ^41–44^ found no impact of holding the baby on anxiety (Spielberger State-Trait Anxiety Inventor, STAI) or depression (CES-D), although there were differences in secondary outcomes, with significantly lower odds of stomach problems for mothers with stillbirths at 28–37 weeks, and lower odds of headache or sleep problems for those with stillbirths after 37 weeks. While not emphasised in the paper, it should be noted that the long list of other assessed symptoms (including panic attacks, backache, fatigue, appetite, patience, self-confidence) were not significantly different in the sample of 314 mothers.

Of the studies that did find significant effects on mental health measures, one^38^ reported significantly decreased odds (OR 0.17, 95% CI 0.05 to 0.56) of post-traumatic stress symptoms at the clinical case level related to holding the baby compared with not, after adjustment for maternal age, parity and induced abortion prior to stillbirth. Another study^22^ found a significant decrease in the odds of anxiety (OR 0.68, 95% CI 0.49 to 0.95) for women who were not currently pregnant, and no significant impact on depression (OR 0.72, 95% CI 0.90 to 5.06) after adjustment for gestation of stillbirth and the time since stillbirth. Conversely, this study found that, for women who were pregnant at the time of survey, odds of anxiety (OR 3.79, 95% CI 1.42 to 10.1) were higher for those who held their baby, again with no significant association with depression (OR 2.13, 95% CI 0.90 to 5.06), after adjustment^22^ in the sample of 2292 women.

Hughes and co-authors^2^ ^3^ ^20^ ^39^ found that, for depression, those who held their stillborn baby had increased odds of an EPDS score above 14 at time 1 (while currently pregnant), although no significant difference was found for mean EPDS score at time 1 or Beck Depression Inventory score at time 2 (1 year after subsequent live birth). For anxiety, continuous state anxiety on the STAI was higher (with a moderate effect size, d=0.51, 95% CI 0.01 to 1.00) at time 1 (currently pregnant) for those who held their infant, but scores at time 2 (1 year after live birth) were not significantly different between those who held and did not hold their baby. PTSD symptoms (from diagnostic interviews) were significantly higher at times 1, 2 and 3 (7–10 years after stillbirth) for women who held their stillborn infant, with moderate to large effect sizes calculated (standardised mean difference ranged from 0.59 to 1.0). These comparisons were not adjusted for potential differences between the groups. The effects of two covariates, socioeconomic status and time since the stillbirth, were reported in the text as reducing the effect on depression at time 1 such that it was no longer significant. Data were not reported after adjustments so reductions in effect sizes could not be calculated. Hughes co-authors^2^ ^3^ ^20^ ^39^ also reported that the odds of marital separation following stillbirth were significantly higher for women who held their stillborn infant (OR 4.50, 95% CI 1.23 to 16.49). Again, there was no adjustment for potential confounding factors.

#### Satisfaction

Ratings of satisfaction with the decision to hold the infant were uniform, with all studies that measured this outcome finding higher rates of satisfaction among women who held their stillborn baby (85–99%) compared with those who did not. In addition, Rådestad *et al*^45^ reported that while holding their stillborn infant, women retrospectively reported feeling warmth (94%) and pride (81%), although this was mixed with insecurity (48%), discomfort (39%) and fear (35%).

### Other contact/memory-making activities

#### Mental health and well-being

Memory-making activities were not generally associated with a significant difference in mental health and well-being outcomes (table 4). Bennett *et al*^36^ reported no significant impact of taking pictures of the infant, and Cacciatore *et al*^22^ reported no significant effect of dressing or washing the baby. Fink^47^ found no significant association between having hand/footprints taken, taking pictures of the baby, or taking a lock of hair, and postnatal grief scores, although there was a small association between creating and having a memory box, and lower grief scores. Gravensteen *et al*^38^ found no association between having an arranged memorial and post-traumatic stress symptoms. Rådestad and co-authors^21^ ^41–44^ reported no difference between those who: kissed or caressed their baby, dressed their baby, kept a photo of their baby or kept another token of remembrance, and depression, after adjustment for maternal education, employment and marital status. There was, however, a significantly higher risk of depression for women who were not with their stillborn baby as long as they wished (risk ratio (RR)=6.9, 95% CI 2.4 to 19.8).^21^ ^41–44^

#### Satisfaction

Five studies reported on maternal satisfaction with having the additional contact/memory-making activities measured in these studies.^9^ ^22^ ^36^ ^40^ ^46^ The majority of mothers were glad they had engaged in these activities and reported that this was helpful or that they were satisfied with their decision. Where assessed, those who had not had the opportunity to engage in such memory-making activities reported wishing they had been able to do so (table 4).

### Inclusion of moderators and subgroup comparisons

Table 5 summarises whether studies included the subgroups/moderators pre-specified for assessment in this review. Few studies included an assessment of proposed moderators. While studies varied in the timing of outcome assessment (time since the stillbirth), only one study investigated the impact of this timing.^2^ ^3^ ^20^ ^39^ Not all comparisons were reported, however, and the authors simply stated that time since the stillbirth had no effect on any associations, with the exception of third trimester (time 1) depression, which became non-significant when this factor was included in an analysis of covariance.^2^ ^3^ ^20^ ^39^ Women's pregnancy status at outcome assessment varied between studies, and was assessed in subgroup analyses by one study, with results reported split by this factor.^22^ No studies provided an analysis of the influence of a subsequent live birth. Cacciatore *et al*^22^ reported that mothers whose babies had congenital anomalies were less likely to see or hold their babies, but that congenital anomalies did not have an effect on any reported analyses and so were not included in results reported in the paper. A number of studies found differential effects of holding the baby according to the gestation of the stillbirth, reported in Results section (table 3).

**Table 5:** Summary of included moderators/subgroup comparisons

Study ID	Time since stillbirth	Women pregnant at outcome assessment	Subsequent live birth/s	Gestation of stillbirth	Time from antepartum death to birth/ or condition of infant	Level of support for contact provided by staff
Bennett *et al*^36^	–	–	–	–	–	–
Blood and Cacciatore^46^	–	–	–	–	–	–
Cacciatore *et al*^22^	–	+	/	/	+	/
Crawley *et al*^37^	–	*	–	–	–	–
Fink 47	–	–	–	–	–	–
Gravensteen *et al*^38^	–	*	–	–	–	–
Hughes *et al*^2^ ^3^ ^20^ ^39^	+	†	‡	–	–	–
Kuti and Ilesanmi^40^	–	–	–	–	–	–
Lasker and Toedter^9^	–	–	–	+	–	–
Rådestad *et al*^21^ ^41–44^	–	/	/	+	/	/
Rådestad *et al*^45^	–	–	–	+	–	–

+, Subgroup comparison or moderation analysis provided. /, variable measured but no subgroup comparison or moderation analysis. –, variable not measured.

*None pregnant at outcome assessment.

†All pregnant at outcome assessment.

‡All women had a subsequent live birth.

Rådestad *et al*^21^ reported that mothers who received staff support to hold the baby (by providing encouragement or formally discussing holding the baby) were more likely to do so. While this comparison was provided, the authors did not assess a potential moderating effect of staff support on outcomes.

## Discussion

### Summary of main results

This review of 11 studies found sparse and conflicting evidence for the impact of holding the stillborn baby on mental health and well-being outcomes. Study quality was generally poor, particularly in sample representativeness, and the adequacy of confounder identification and adjustment. There were mixed results for the impact of holding the stillborn baby on mental health and well-being. One study found no significant effects,^36^ and two other studies reported no impact on depression after adjustment for confounders.^21^ ^22^ ^41–44^ Conflicting effects were found for anxiety and post-traumatic stress, with one study reporting increased odds associated with holding the baby,^2^ ^3^ ^20^ ^39^ one reporting decreased odds of anxiety and post-traumatic stress,^38^ and one reporting a decrease for those not currently pregnant but an increase for those currently pregnant.^22^ Significant selection bias and confounding is likely in many of the reported results and effect sizes should therefore be interpreted with caution. Included studies were heterogeneous in approach, and many failed to provide adequate comparisons for the primary intervention and outcomes. Consistent with qualitative evidence,^7^ ^11–13^ studies consistently found that women were satisfied with their decision to hold their stillborn baby. Given women's clear, high levels of satisfaction with the decision to hold their baby, the equipoise that would be required to conduct randomised trials must be justified. While few studies evaluated the same memory-making activities, current evidence suggests no significant positive or negative impacts of activities such as collecting hand/footprints or taking photos on mental health and well-being outcomes, but women were satisfied with their decision to engage in these activities.

### Completeness and availability of evidence

As noted previously, sparse evidence exists for the impact of contact with the stillborn infant on parental outcomes.^14^ As stillbirth is a relatively rare event, it is unsurprising that sample sizes were small. Most studies also failed to be representative of the wider stillbirth population, with many using volunteers recruited through stillbirth organisation websites or strict eligibility criteria^2^ ^3^ ^20^ ^39^ that excluded many women. Six moderators/subgroups that may have a significant impact on the relationship between contact and outcomes were explored in this review; few studies measured, and even fewer investigated the effects of these factors.

Support for contact with the stillborn baby was not assessed as a moderator in any studies, but may be important in influencing the nature of this contact and its impact. Furthermore, broader cultural support for holding or other memory-making activities may also be important. Most studies were conducted in high-income country contexts, and results may not translate cross-culturally where expectations and traditions around mourning, for example, differ.

Beyond the moderators discussed, no studies addressed individual factors that may also influence the impact of holding or other memory-making activities on parental outcomes. No studies reported on outcomes for partners, and while one study investigated impacts on relationship satisfaction, no studies looked at whether women held their baby with their partner or alone, or if partners and family were supportive.^2^ ^3^ ^20^ ^39^

### Quality of evidence

This review highlighted a number of difficulties and weaknesses in attempting to provide an adequate assessment of the impact of infant contact in absence of clinical trial evidence. Recruiting a representative and sufficiently large sample for a relatively rare event presents a challenge. The quality of the evidence suffered particularly due to a lack of investigation of characteristics that differed between those who held and did not hold their baby. This led to inadequate confounder adjustment, as it is likely that many factors systematically differed between the two groups.^22^ ^21^ As random allocation is not possible in these observational studies, greater attention to potential confounders would significantly improve the validity of results presented, and eliminate some alternative explanations for relationships found and the differences between studies.^20^ ^22^ A stronger theoretical basis for the proposed associations and explication of the pathways through which contact with the infant influences outcomes would provide hypotheses that could be tested and is likely to aid in the assessment of key moderators and potential confounders.

Few studies investigated the role of hypothesised moderators. Future attention to these factors may highlight the conditions under which contact with the stillborn infant may be beneficial or harmful to parents, and provide clearer guidance for clinical practice. In particular, the role of staff and the way in which the baby is presented has significant implications for clinical practice.^21^

However, while greater attention to confounder adjustment would greatly improve future studies, it should be noted that, even if groups were found to be comparable on measured characteristics or adjustment was made for many relevant variables, it remains possible that differences identified may be attributable to other unknown factors differing between the groups.^34^

There were a number of strengths in the evidence identified. Cross-sectional designs meant that outcome data were generally complete, and the two longitudinal studies identified had high retention rates.^3^ ^9^ ^20^

Given the salience of stillbirth and surrounding events to women and partners, and evidence of the reliability of women's self-report regarding events around birth,^67–69^ information collected from women probably represents the most effective and accurate estimate of whether or not women held their stillborn baby. As noted in the results, no studies clearly defined and assessed the way the infant was held (eg, skin-to-skin, timing or duration). These characteristics have been suggested to influence outcomes for women who held their live newborn, with some evidence of a dose–response effect,^66^ and it is unclear if this is the case when the baby has died. Future investigation of these factors may be important in clarifying effects, however, long-term recall regarding the duration or timing of holding may be less accurate.^69^

While no studies used clinical diagnosis of mental health problems as an outcome, this may not be feasible in studies with large sample sizes. A strength of the included studies was that most used validated, well-known measures of mental health outcomes. This aided comparability, although the wide variety of such scales meant few studies used the same instrument, which made comparing outcomes more challenging, with each included scale having associated strengths and weaknesses. While these scales are useful for comparability across studies, the use of clinical psychological symptomatology could be questioned. These measures are typically designed to assess pathology, and it could be argued that more subtle changes in quality of life, grief or more general well-being may also be important to consider. Validated measures of quality of life may represent a useful additional outcome in future studies.

There is no evidence regarding the impact of holding the stillborn infant on fathers and partners. Evidence suggests that they are also highly affected by a stillbirth,^12^ ^70^ and future work is needed to address this deficit.

### Strengths and weaknesses of the review

This review is the first to collate, summarise and appraise the available evidence of the impact of holding the stillborn baby on parental mental health, well-being and satisfaction. While assessment of risk of bias is controversial for the evaluation of non-randomised designs, the use of a robust quality appraisal framework based on established risk of bias assessment for randomised trials and critical appraisal tools for non-randomised studies^30^ ^34^ ^65^ was a significant strength of the present review. Reviews of non-randomised studies have often failed to provide thorough study quality appraisal, which is essential in determining the rigour of included studies and the amount of confidence readers may place in the effect sizes presented. While thorough quality appraisal was provided, consensus on the items for appraisal required for non-randomised studies is yet to be reached.^29^ ^30^ ^65^

This review included a comprehensive search of past literature, and searches of peer-reviewed and grey literature, with database searching supplemented by hand-searching references and contacting experts. Two foreign language papers were identified, but resources for translation were unavailable, so it is unclear if these papers would have been eligible for inclusion.

## Conclusions

In seeking to provide women and their partners with the best available evidence to make informed decisions about having contact with their stillborn baby, the present review found no clear evidence for the impact of holding the stillborn infant on mental health and well-being outcomes in either direction. The review does support qualitative evidence that suggests this contact is valued by women and that they are retrospectively satisfied with their decisions to do so. Reliable data were sparse regarding other memory-making activities, although present evidence suggests there may be no effect of these activities on short-term or long-term mental health and well-being, and that parents are typically happy with their decision to participate in these activities.

Important hypothesised moderators of these effects are yet to be adequately tested. In particular, the condition of the baby, gestation at stillbirth, and the role of care provider support and the way the baby is presented may all be important moderators of the impact that contact with the baby has on outcomes.

Findings from this review suggest that guideline recommendations suggesting women should or should not be encouraged to hold their stillborn infant^15–19^ do not reflect current evidence, which provides no clear guidance for practice. Evidence does suggest women have been satisfied with their decision to hold their baby. There is no current evidence for the impact of contact with the stillborn baby on outcomes for partners. Further research in this area is needed coupled with research to provide guidance regarding partners’ contact with their stillborn infant.

## References

[1] Flenady V, Koopmans L, Middleton P Major risk factors for stillbirth in high-income countries: a systematic review and meta-analysis. Lancet 2011;377:1331–40. 10.1016/S0140-6736(10)62233-7 21496916

[2] Turton P, Hughes P, Evans C Incidence, correlates and predictors of post-traumatic stress disorder in the pregnancy after stillbirth. Br J Psychiatry 2001;178:556–60. 10.1192/bjp.178.6.556 11388974

[3] Turton P, Evans C, Hughes P Long-term psychosocial sequelae of stillbirth: phase II of a nested case-control cohort study. Arch Womens Ment Health 2009;12:35–41. 10.1007/s00737-008-0040-7 19137447

[4] Harper M, O'Connor RC, O'Carroll RE Increased mortality in parents bereaved in the first year of their child's life. BMJ Support Palliat Care 2011;1:306–9. 10.1136/bmjspcare-2011-000025 24653475

[5] Kelley MC, Trinidad SB Silent loss and the clinical encounter: parents’ and physicians’ experiences of stillbirth—a qualitative analysis. BMC Pregnancy Childbirth 2012;12:137 10.1186/1471-2393-12-137 23181615 PMC3533522

[6] Mills TA, Ricklesford C, Cooke A Parents’ experiences and expectations of care in pregnancy after stillbirth or neonatal death: a metasynthesis. BJOG 2014;121:943–50. 10.1111/1471-0528.12656 24589119

[7] Downe S, Schmidt E, Kingdon C Bereaved parents’ experience of stillbirth in UK hospitals: a qualitative interview study. BMJ Open 2013;3:pii: e002237 10.1136/bmjopen-2012-002237 PMC3586079 23418300

[8] Lewis E The management of stillbirth coping with an unreality. Lancet 1976;308:619–20. 10.1016/S0140-6736(76)90680-2 61354

[9] Lasker JN, Toedter LJ Satisfaction with hospital care and interventions after pregnancy loss. Death Stud 1994;18:41–64. 10.1080/07481189408252642 10184042

[10] Cunningham KA Holding a stillborn baby: does the existing evidence help us provide guidance? Med J Aust 2012;196:558 10.5694/mja11.11417 22621140

[11] Gold KJ, Dalton VK, Schwenk TL Hospital care for parents after perinatal death. Obstet Gynecol 2007;109:1156–66. 10.1097/01.AOG.0000259317.55726.df 17470598

[12] Cacciatore J, Erlandsson K, Rådestad I Fatherhood and suffering: a qualitative exploration of Swedish men's experiences of care after the death of a baby. Int J Nurs Stud 2013;50:664–70. 10.1016/j.ijnurstu.2012.10.014 23177900

[13] Ryninks K, Roberts-Collins C, McKenzie-McHarg K Mothers’ experience of their contact with their stillborn infant: an interpretative phenomenological analysis. BMC Pregnancy Childbirth 2014;14:203 10.1186/1471-2393-14-203 24923242 PMC4062775

[14] Koopmans L, Wilson T, Cacciatore J Support for mothers, fathers and families after perinatal death. The Cochrane Library, 2013. 10.1002/14651858.CD000452.pub3 PMC7086381 23784865

[15] National Institute for Health and Clinical Excellence (NICE). Antental and postnatal mental health. London: NICE, 2007.

[16] National Institute for Health and Clinical Excellence (NICE). Routine postnatal care of women and their babies. London: NICE, 2006. 31825570

[17] National Institute for Health and Clinical Excellence (NICE). Antenatal and postnatal mental health: clinical management and service guidance. London: NICE uidelines[CG192], 2014. 31990493

[18] Royal College of Obstetricians and Gynaecologists. The Rcog Green-Top Guideline No.55 Late Intrauterine Fetal Death and Stillbirth. NHS Evidence, 2010.

[19] Queensland Maternity and Neonatal Clinical Guidelines. “Stillbirth Care.” edited by Queensland Maternity and Neonatal Clinical Guidelines Program. Queensland: Queensland Government, 2011.

[20] Hughes P, Turton P, Hopper E Assessment of guidelines for good practice in psychosocial care of mothers after stillbirth: a cohort study. Lancet 2002;360:114–8. 10.1016/S0140-6736(02)09410-2 12126820

[21] Rådestad I, Surkan PJ, Steineck G Long-term outcomes for mothers who have or have not held their stillborn baby. Midwifery 2009;25:422–9. 10.1016/j.midw.2007.03.005 18069101

[22] Cacciatore J, Rådestad I, Frederik Frøen J Effects of contact with stillborn babies on maternal anxiety and depression. Birth 2008;35:313–20. 10.1111/j.1523-536X.2008.00258.x 19036044

[23] Geller PA, Psaros C, Kornfield SL Satisfaction with pregnancy loss aftercare: are women getting what they want? Arch Womens Ment Health 2010;13:111–24. 10.1007/s00737-010-0147-5 20177721

[24] Erlandsson K, Warland J, Cacciatore J Seeing and holding a stillborn baby: mothers’ feelings in relation to how their babies were presented to them after birth—findings from an online questionnaire. Midwifery 2013;29:246–50. 10.1016/j.midw.2012.01.007 22410169

[25] Stroup DF, Berlin JA, Morton SC Meta-analysis of observational studies in epidemiology: a proposal for reporting. JAMA 2000;283:2008–12. 10.1001/jama.283.15.2008 10789670

[26] Moher D, Liberati A, Tetzlaff J Preferred reporting items for systematic reviews and meta-analyses: the PRISMA statement. Ann Intern Med 2009;151:264–9. 10.7326/0003-4819-151-4-200908180-00135 19622511

[27] Lawn JE, Blencowe H, Pattinson R Stillbirths: Where? When? Why? How to make the data count? Lancet 2011;377:1448–63. 10.1016/S0140-6736(10)62187-3 21496911

[28] Redshaw M, Rowe R, Henderson J Listening to Parents after stillbirth or the death of their baby after birth. Oxford: National Perinatal Epidemiology Unit, 2014.

[29] Higgins JP, Green S Cochrane handbook for systematic reviews of interventions. Wiley Online Library, 2011.

[30] Sanderson S, Tatt ID, Higgins JP Tools for assessing quality and susceptibility to bias in observational studies in epidemiology: a systematic review and annotated bibliography. Int J Epidemiol 2007;36:666–76. 10.1093/ije/dym018 17470488

[31] von Elm E, Altman DG, Egger M The Strengthening the Reporting of Observational Studies in Epidemiology (STROBE) statement: guidelines for reporting observational studies. Prev Med 2007;45:247–51. 10.1016/j.ypmed.2007.08.012 17950122

[32] Critical Appraisal Skills Program (CASP). 12 Questions to help you make sense of a cohort study. Oxford: CASP, 2013 http://www.casp-uk.net/#!casp-tools-checklists/c18f8 (accessed Jun 2014).

[33] Critical Appraisal Skills Program (CASP). 11 Questions to help you make sense of a case control study. Oxford: CASP, 2013 http://www.casp-uk.net/#!casp-tools-checklists/c18f8 (accessed Jun 2014).

[34] Wells GA, Shea B, O'Connell D Quality assessment scales for observational studies. Ottawa: Ottawa Health Research Institute, 2004.

[35] Shadish WR, Clark M, Steiner PM Can nonrandomized experiments yield accurate answers? A randomized experiment comparing random and nonrandom assignments. J Am Stat Assoc 2008;103:1334–44. 10.1198/016214508000000733

[36] Bennett SM, Litz BT, Maguen S An exploratory study of the psychological impact and clinical care of perinatal loss. J Loss Trauma 2008;13:485–510. 10.1080/15325020802171268

[37] Crawley R, Lomax S, Ayers S Recovering from stillbirth: the effects of making and sharing memories on maternal mental health. J Reprod Infant Psychol 2013;31:195–207. 10.1080/02646838.2013.795216

[38] Gravensteen IK, Helgadóttir LB, Jacobsen EM Women's experiences in relation to stillbirth and risk factors for long-term post-traumatic stress symptoms: a retrospective study. BMJ Open 2013;3:e003323 10.1136/bmjopen-2013-003323 PMC3808779 24154514

[39] Hughes PM *The effect of previous stillbirth on maternal mental health in the subsequent pregnancy and on patterns of attachment in mother and next-born infant*[M.D.]. Ann Arbor: University of London, St. George's Hospital Medical School (United Kingdom), 2003.

[40] Kuti O, Ilesanmi CE Experiences and needs of Nigerian women after stillbirth. Int J Gynecol Obstet 2011;113:205–7. 10.1016/j.ijgo.2010.11.025 21458808

[41] Rådestad I, Nordin C, Steineck G Stillbirth is no longer managed as a nonevent: a nationwide study in Sweden. Birth 1996;23:209–15. 10.1111/j.1523-536X.1996.tb00496.x 9086957

[42] Rådestad I, Steineck G, Nordin C Psychological complications after stillbirth—influence of memories and immediate management: population based study. BMJ 1996;312:1505–8. 10.1136/bmj.312.7045.1505 8646141 PMC2351280

[43] Surkan PJ, Rådestad I, Cnattingius S Events after stillbirth in relation to maternal depressive symptoms: a brief report. Birth 2008;35:153–7. 10.1111/j.1523-536X.2008.00229.x 18507587

[44] Rådestad I Stillbirth: care and long-term psychological effects. Br J Midwifery 2001;9:474–80. 10.12968/bjom.2001.9.8.7931

[45] Rådestad I, Saflund K, Wredling R Holding a stillborn baby: mothers’ feelings of tenderness and grief. Br J Midwifery 2009;17:178–80. 10.12968/bjom.2009.17.3.40082

[46] Blood C, Cacciatore J Parental grief and memento mori photography: narrative, meaning, culture, and context. Death Stud 2014;38:224–33. 10.1080/07481187.2013.788584 24524585

[47] Fink PL The relationships between perinatal loss interventions at the time of stillbirth and maternal grief. West Virginia University, 2012.

[48] Wilson DB Practical meta-analysis effect sixe calculator. http://www.campbellcollaboration.org/escalc/html/EffectSizeCalculator-Home.php

[49] Wilson T, Boyle F, Ware R Holding a stillborn baby: the view from a specialist perinatal bereavement service. J Paediatr Child Health 2013;49:25. 10.1111/ajo.12327 26129981

[50] Toedter LJ, Lasker JN, Alhadeff JM The Perinatal Grief Scale: development and initial validation. Am J Orthopsychiatry 1988;58:435 10.1111/j.1939-0025.1988.tb01604.x 3407734

[51] Prigerson HG, Maciejewski PK, Reynolds CF III Inventory of Complicated Grief: a scale to measure maladaptive symptoms of loss. Psychiatry Res 1995;59:65–79. 10.1016/0165-1781(95)02757-2 8771222

[52] Weathers FW, Litz BT, Herman DS, eds The PTSD Checklist (PCL): reliability, validity, and diagnostic utility. Annual Convention of the International Society for Traumatic Stress Studies; International Society for Traumatic Stress Studies San Antonio, 1993.

[53] Derogatis LR BSI 18, Brief Symptom Inventory 18: administration, scoring and procedures manual. NCS Pearson, Incorporated, 2001.

[54] Derogatis LR, Lipman RS, Rickels K The Hopkins Symptom Checklist (HSCL): a self-report symptom inventory. Behav Sci 1974;19:1–15. 10.1002/bs.3830190102 4808738

[55] Lovibond PF, Lovibond SH The structure of negative emotional states: comparison of the Depression Anxiety Stress Scales (DASS) with the Beck Depression and Anxiety Inventories. Behav Res Ther 1995;33:335–43. 10.1016/0005-7967(94)00075-U 7726811

[56] Foa EB, Riggs DS, Dancu CV Reliability and validity of a brief instrument for assessing post-traumatic stress disorder. J Trauma Stress 1993;6:459–73. 10.1002/jts.2490060405

[57] Horowitz M, Wilner N, Alvarez W Impact of Event Scale: a measure of subjective stress. Psychosom Med 1979;41:209–18. 10.1097/00006842-197905000-00004 472086

[58] Goldberg D, Williams P General health questionnaire (GHQ). Swindon, Wiltshire, UK: NFER Nelson, 2000.

[59] Radloff LS The CES-D scale a self-report depression scale for research in the general population. Appl Psychol Meas 1977;1:385–401. 10.1177/014662167700100306

[60] Cox JL, Holden JM, Sagovsky R Detection of postnatal depression. Development of the 10-item Edinburgh Postnatal Depression Scale. Br J Psychiatry 1987;150:782–6. 10.1192/bjp.150.6.782 3651732

[61] Spielberger CD, Gorsuch RL, Lushene RE *Manual for the state-trait anxiety inventory* Palo Alto, CA: Consulting Psychologists Press, 1983.

[62] Beck AT, Ward CH, Mendelson M An inventory for measuring depression. Arch Gen Psychiatry 1961;4:561–71. 10.1001/archpsyc.1961.01710120031004 13688369

[63] Watson CG, Juba MP, Manifold V The PTSD interview: rationale, description, reliability, and concurrent validity of a DSM-III-based technique. J Clin Psychol 1991;47:179–88. 10.1002/1097-4679(199103)47:2<179::AID-JCLP2270470202>3.0.CO;2-P 2030122

[64] WHO. ICD-10: international statistical classification of diseases and related health problems—instruction manual. Geneva, Switzerland: World Health Organization, 2004.

[65] Higgins J, Ramsay C, Reeves BC Issues relating to study design and risk of bias when including non-randomized studies in systematic reviews on the effects of interventions. Res Synth Methods 2013;4:12–25. 10.1002/jrsm.1056 26053536

[66] Redshaw M, Hennegan J, Kruske S Holding the baby: early mother–infant contact after childbirth and outcomes. Midwifery 2014;30:e177–e87. 10.1016/j.midw.2014.02.003 24680108

[67] Poulsen G, Kurinczuk JJ, Wolke D Accurate reporting of expected delivery date by mothers 9 months after birth. J Clin Epidemiol 2011;64:1444–50. 10.1016/j.jclinepi.2011.03.007 21684117

[68] Quigley M, Hockley C, Davidson L Agreement between hospital records and maternal recall of mode of delivery: evidence from 12 391 deliveries in the UK Millennium Cohort Study. BJOG 2007;114:195–200. 10.1111/j.1471-0528.2006.01203.x 17166217

[69] Olson JE, Shu XO, Ross JA Medical record validation of maternally reported birth characteristics and pregnancy-related events: a report from the Children's Cancer Group. Am J Epidemiol 1997;145:58–67. 10.1093/oxfordjournals.aje.a009032 8982023

[70] Badenhorst W, Riches S, Turton P The psychological effects of stillbirth and neonatal death on fathers: systematic review. J Psychosom Obstet Gynecol 2006;27:245–56. 10.1080/01674820600870327 17225626

